# Supplementary material for: Improving wheat grain composition for human health by constructing a QTL atlas for essential minerals
Source: Commun Biol. 2024 Aug 15;7:1001. doi: 10.1038/s42003-024-06692-7 (PMC11327371; doi:10.1038/s42003-024-06692-7)
Supplement: Supplementary file 2 — Supplementary Material [file 42003_2024_6692_MOESM2_ESM.pdf]

Supplementary Materials for:

Improving wheat grain composition for human health by constructing a QTL atlas for essential minerals.

Petros P. Sigalas[1*], Peter R. Shewry[1*], Andrew Riche[1], Luzie Wingen[2], Cong Feng[3], Ajay Siluveru[2], Noam Chayut[2], Amanda Burridge[4], Cristobal Uauy[2], March Castle[1], Saroj Parmar[1], Charlie Philp[2], David Steele[1], Simon Orford[2], Michelle Leverington-Waite[2], Shifeng Cheng[3], Simon Griffiths[2] and Malcolm J. Hawkesford[1**]

[1]*Rothamsted Research, Harpenden, Hertfordshire, ALF 2JQ, UK*
[2]*John Innes Centre, Norwich, Norfolk, NR4 7UH, UK*
[3]*Shenzhen Branch, Guangdong Laboratory for Lingnan Modern Agriculture, Genome Analysis Laboratory of the Ministry of Agriculture, Agricultural Genomics Institute at Shenzhen, Chinese Academy of Agricultural Sciences, Shenzhen 518124, China*
[4]*School of Biological Sciences, University of Bristol, Bristol, BS8 1UD, UK*

*These authors contributed equally.
**Corresponding author (email malcolm.hawkesford@rothamsted.ac.uk).

Supplementary Material List

Supplementary Fig. 1
Supplementary Fig. 2
Supplementary Table 1
Supplementary Table 2

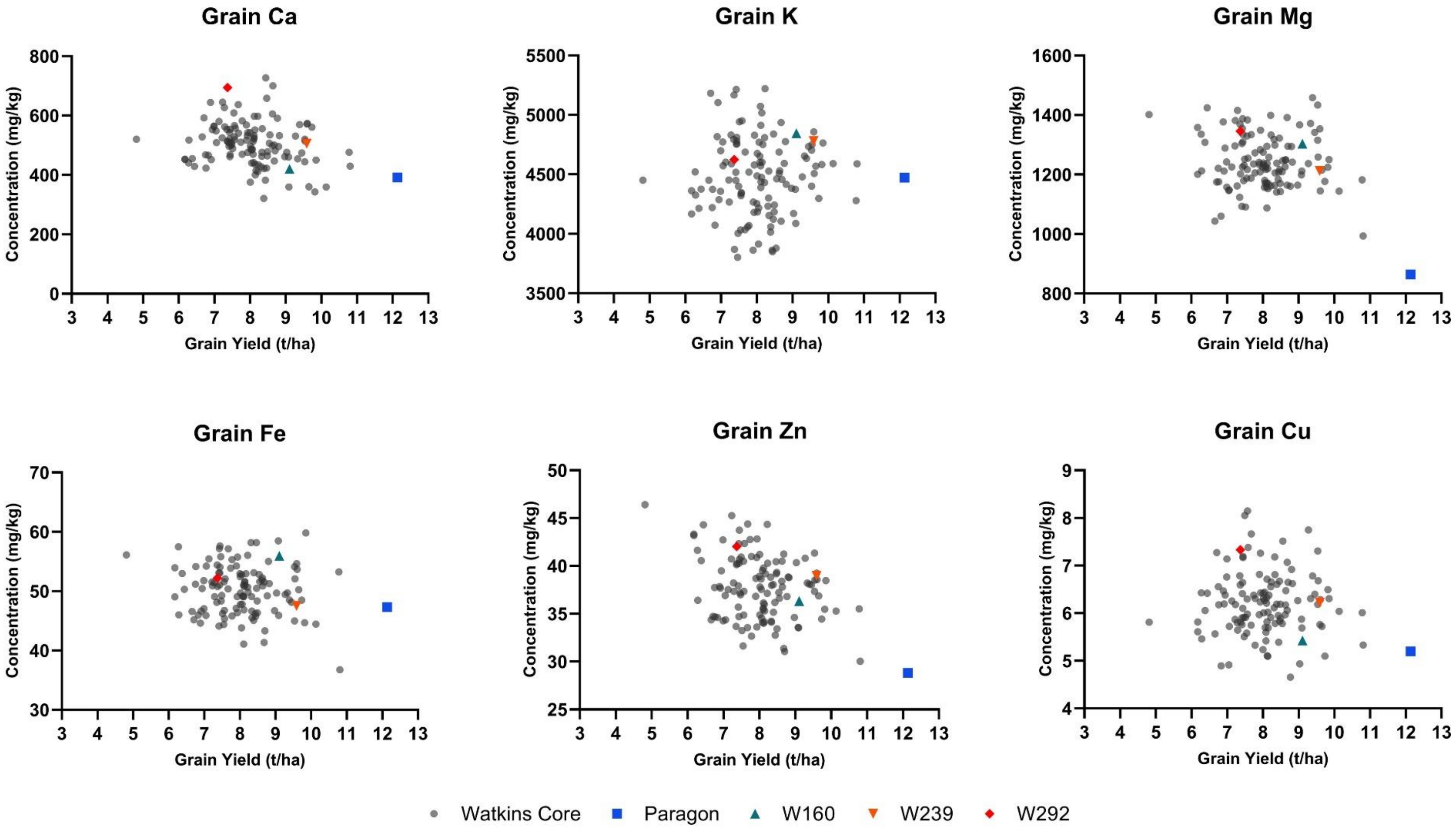

Supplementary Fig. 1: Relationship between grain mineral concentration and grain yield in A. E. Watkins landraces and cv. Paragon - This figure illustrates the relationship between the grain mineral concentration (Ca, K, Mg, Fe, Zn, and Cu) and the grain yield of 119 Watkins landraces from the core A.E. Watkins collection and cv. Paragon. These were grown in randomised block design with 3 replicates at Rothamsted Research for four successive years, with the data representing the mean values over the four-year period (2011-2014) (n=12). The three selected Watkins landraces, which were used as parents for the development of biparental segregating populations, along with cv. Paragon, are depicted in distinct colours. Plots were created in GraphPad Prism v9.3.1.

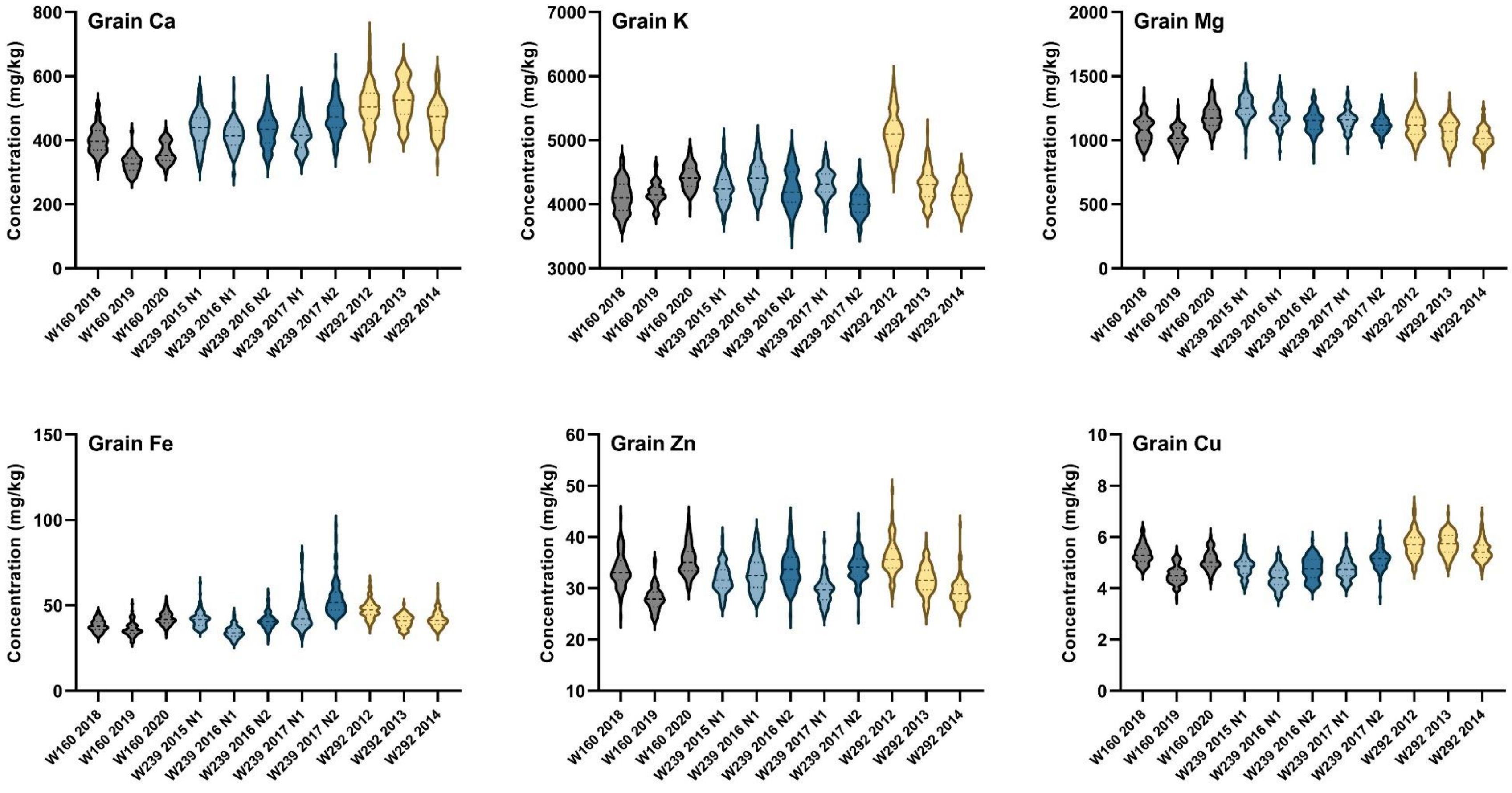

Supplementary Fig. 2: Violin plots of grain mineral concentration in biparental populations across different environments - This figure illustrates the distribution of grain mineral concentration (Ca, K, Mg, Fe, Zn, and Cu) in three biparental populations (Par x W160, Par x W239, Par x W292) across different environments (year, N treatment). Each population comprised 94 F4 recombinant inbred lines. Three biological replicates were included in each trial (n=3) with 1 m x 1 m plot as experimental unit. ANOVA results for each population and year can be found in Supplementary Data 3. Plots were created in GraphPad Prism v9.3.1.

Supplementary Table 1: Landrace parents of the three populations, selected from the A.E Watkins bread wheat landrace collection.

GRU Code	Watkins Code	Abbreviation	Country Code	Country of origin	Growth Habit	Locality	Latitude	Longitude	Ancestral Group [21, 63]
WATDE0021	1190160 - 2	W160	ESP	Spain	spring	Navarre	42.72	-1.67	C7
WATDE0032	1190239 - 1	W239	ESP	Spain	spring	Gallegos, Salamanca	40.85	-6.71	C6
WATDE0038	1190292 - 1	W292	CYP	Cyprus	spring	Paphos district	34.88	32.52	C6

Supplementary Table 2: Information about site soil type, dates of sowing, soil mineral N and N applications rates for each year and population.

Year	Population	Soil type	Drilling date	Soil Mineral N (kg N/ha)	N1 - Low N fertilization (kg N/ha)	N2 - High N fertilization (kg N/ha)	1st N application (50 kg/ha) - Both N1 and N2	2nd N application (100 kg/ha) - N2 only	3rd N application (50 kg/ha) - N2 only	Harvest date
2012	Par x W292	Very slightly flinty clay loam	24-26/10/11	70	NA	200	14/03/2012	13/04/2012	17/05/2012	24/8-8/9/2012
2013	Par x W292	Slightly flinty, very slightly calcareous clay loam to silty clay loam (Ap horizon)	27/2-01/03/13	74	NA	200	15/04/2013	30/04/2013	21/05/2013	29/8-5/9/2013
2014	Par x W292	Very slightly flinty silty clay loam (Ap horizon)	29-30/10/13	71	NA	200	12/03/2014	03/04/2014	24/04/2014	13-21/8/2014
2015	Par x W239	Slightly flinty, very slightly calcareous silty clay loam (ap horizon)	27-28/10/14	40	50	200	16/03/2015	01/04/2015	30/04/2015	7-11/09/2015
2016	Par x W239	Slightly flinty, very slightly calcareous clay loam to silty clay loam (Ap horizon)	16-23/10/15	63	50	200	17/03/2016	08/04/2016	27/04/2016	11-18/08/2016
2017	Par x W239	Very slightly flinty silty clay loam (Ap horizon)	19-20/10/16	43	50	200	09/03/2017	06/04/2017	11/05/2017	15-26/08/2017
2018	Par x W160	Slightly flinty, very slightly calcareous silty clay loam (ap horizon)	14/10/2017	101	NA	200	16/04/2018	23/04/2018	04/05/2018	2-15/08/2018
2019	Par x W160	Slightly flinty, very slightly calcareous clay loam to silty clay loam (Ap horizon)	22/10/2018	103	NA	200	28/02/2019	03/04/2019	15/05/2019	21-24/08/2019
2020	Par x W160	Very slightly flinty silty clay loam (Ap horizon)	21/11/2019	77	NA	200	16/03/2020	14/05/2020	09/06/2020	10-11/08/2020
