# Supplementary material for: Improving wheat grain composition for human health by constructing a QTL atlas for essential minerals
Source: Commun Biol. 2024 Aug 15;7:1001. doi: 10.1038/s42003-024-06692-7 (PMC11327371; doi:10.1038/s42003-024-06692-7)
Supplement: Supplementary file 3 — Description of Additional Supplementary Files [file 42003_2024_6692_MOESM3_ESM.pdf]

Description of Additional Supplementary Files

File name: Supplementary Data 1

Description: Crop ontology terms list.

File name: Supplementary Data 2

Description: Comprehensive QTL analysis results for populations Par x W160, Par x W239, and Par x W292.

File name: Supplementary Data 3

Description: ANOVA table for grain mineral concentration across different populations and years.

File name: Supplementary Data 4

Description: Correlation of grain mineral concentrations and plant height, straw biomass, above ground biomass, grain yield, harvest index and thousand grain weight in populations Par x W160, Par x W239, and Par x W292.

File name: Supplementary Data 5

Description: Summary of QTL analysis results and list of surrounding genes of selected QTLs.

File name: Supplementary Data 6

Description: Grain mineral content and characteristics of selected TILLING mutant lines

File name: Supplementary Data 7

Description: Heritability values for grain mineral concentration and other yield related traits.
